# The therapeutic effect of chlorogenic acid against *Staphylococcus aureus* infection through sortase A inhibition

**DOI:** 10.3389/fmicb.2015.01031

**Published:** 2015-10-16

**Authors:** Lin Wang, Chongwei Bi, Hongjun Cai, Bingrun Liu, Xiaobo Zhong, Xuming Deng, Tiedong Wang, Hua Xiang, Xiaodi Niu, Dacheng Wang

**Affiliations:** ^1^Key Laboratory of Zoonosis Research, Ministry of Education/Institute of Zoonosis/College of Veterinary Medicine, Jilin UniversityChangchun, China; ^2^College of Animal Science, Jilin UniversityChangchun, China; ^3^The College of Animal Science and Technology, Jilin Agricultural UniversityChangchun, China; ^4^Key Laboratory of Zoonosis Research, Ministry of Education/Department of Food Quality and Safety/College of Veterinary Medicine, Jilin UniversityChangchun, China

**Keywords:** sortase A, *Staphylococcus aureus*, chlorogenic acid, renal abscess, binding site, inhibitor

## Abstract

The emergence and wide spread of multi-drug resistant *Staphylococcus aureus* (*S. aureus*) requires the development of new therapeutic agents with alternative modes of action. Anti-virulence strategies are hoped to meet that need. Sortase A (SrtA) has attracted great interest as a potential drug target to treat infections caused by *S. aureus*, as many of the surface proteins displayed by SrtA function as virulence factors by mediating bacterial adhesion to specific organ tissues, invasion of host cells, and evasion of the host-immune responses. It has been suggested that inhibitors of SrtA might be promising candidates for the treatment and/or prevention of *S. aureus* infections. In this study, we report that chlorogenic acid (CHA), a natural compound that lacks significant anti-*S. aureus* activity, inhibit the activity of SrtA *in vitro* (IC_50_ = 33.86 ± 5.55 μg/ml) and the binding of *S. aureus* to fibrinogen (Fg). Using molecular dynamics simulations and mutagenesis assays, we further demonstrate that CHA binds to the binding sites of C184 and G192 in the SrtA. *In vivo* studies demonstrated that CHA prevent mice from *S. aureus*-induced renal abscess, resulting in a significant survival advantage. These findings indicate that CHA is a promising therapeutic compound against SrtA during *S. aureus* infections.

## Introduction

*Staphylococcus aureus* (*S. aureus*) is an opportunistic pathogen which produces a wide spectrum of diseases, ranging from minor skin infections and soft tissue infections to bacteraemia and toxic shock syndrome. Bacteraemia frequently leads to infective endocarditis, osteomyelitis, septic arthritis, and metastatic abscess formation (Lowy, 1998; Ippolito et al., 2010). *S. aureus* is recognized as a prominent pathogen and has developed a wide range of resistance to antibiotics (such as methicillin and vancomycin), as well as causing severe clinical complications and poor outcomes (Vincent et al., 2006; Naber, 2009), making treatment options difficult. The prevalence of the antibiotic -resistant *S. aureus* isolates indicates the need for alternative therapy to treat these infections (Ton-That and Schneewind, 1999). Recently, anti-virulence strategies to combat bacteria-mediated infections have gained great interest.

The virulence factors of pathogens play a key role in the establishment of an infection. The ability of *S. aureus* to cause disease has been generally attributed to two classes of virulence factors: cell surface proteins and extracellular protein toxins. The surface proteins, such as protein A, clumping factor and fibronectin-binding proteins, mediate adhesion to host endothelial tissues and evasion of host complement proteins and immunoglobulin (Scott and Barnett, 2006). These virulence-associated surface proteins are covalently anchored to bacterial cell wall peptidoglycans through a general sorting mechanism catalyzed by a superfamily of membrane-associated transpeptidases termed sortases (Maresso and Schneewind, 2008). The sortase A (SrtA) isoform plays a critical role in the pathological effects of *S. aureus* (Maresso and Schneewind, 2008). Earlier work identified several *srtA* mutants defective for anchoring ∼19 surface proteins with LPXTG sorting signals to the cell wall envelope (Mazmanian et al., 2002). These *srtA* mutants have decreased virulence and cannot cause lethal sepsis or form abscesses in mouse models of staphylococcal disease (Mazmanian et al., 2000; Cheng et al., 2009; McAdow et al., 2011). Disrupting the display of these proteins by blocking the activity of SrtA using an inhibitor could therefore effectively reduce bacterial virulence and thus promote bacterial clearance by the host (Mazmanian et al., 2000). Attractively, SrtA inhibitors may also be less likely to induce selective pressures that lead to drug resistance, as these strains do not exhibit impaired growth in culture medium outside of their human host (Mazmanian et al., 1999). Therefore, SrtA is an attractive target to attenuate virulence and hamper *S. aureus* infections.

Shortly after the discovery of SrtA, many studies toward finding a potent inhibitor have been conducted over the past decade. Multiple classes of molecules able to inhibit sortase has been identified, including non-specific inhibitors, peptide-analogs, natural products, and synthetic small molecules, which is the first step in the development of chemotherapeutics to be used in the clinic (reviewed in Clancy et al., 2010; Zhang et al., 2014).

**FIGURE 1:**
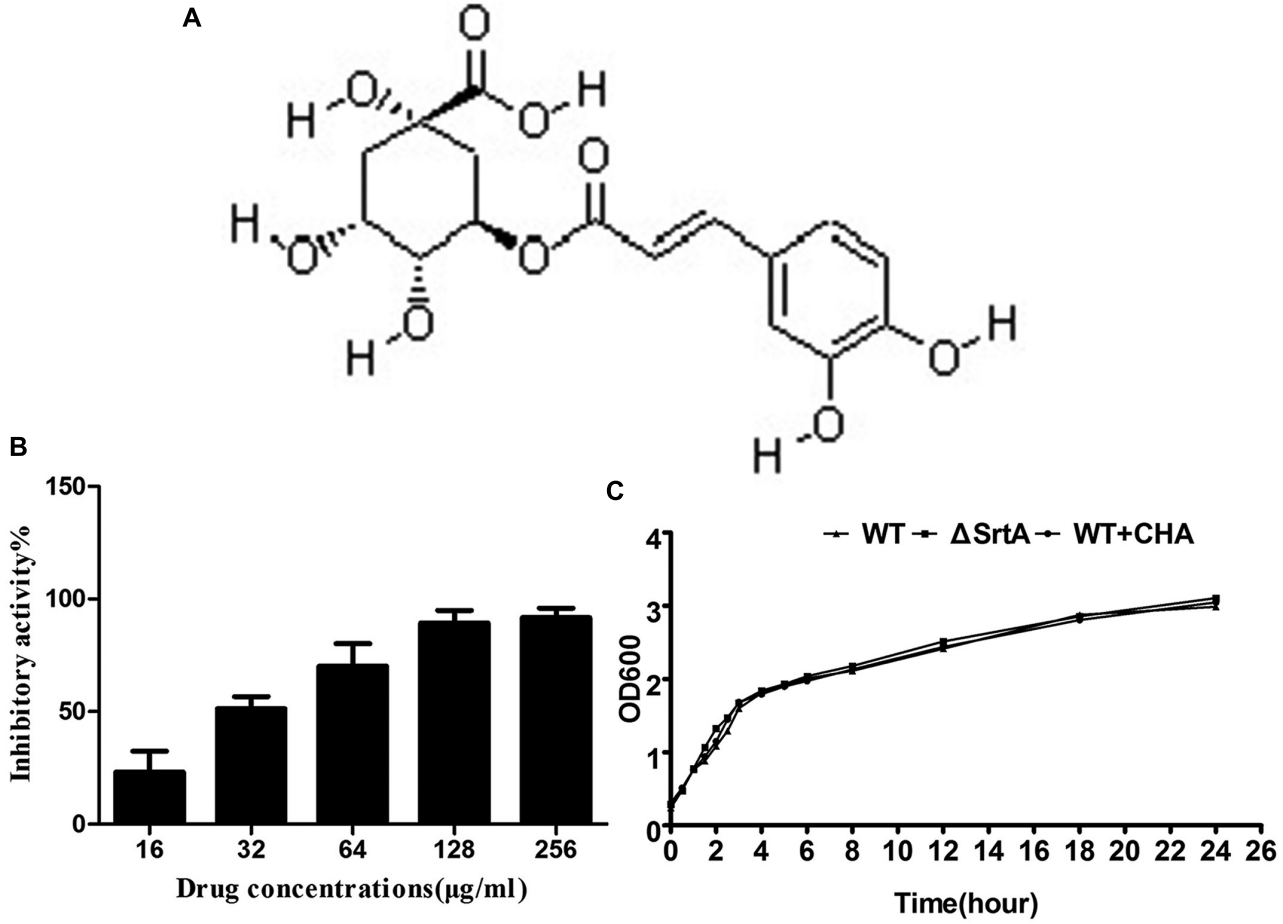
**(A)** Structure of chlorogenic acid (CHA). **(B)** Inhibitory effects of CHA (different concentrations) against SrtA from *Staphylococcus aureus* Newman D2C *in vitro*. **(C)** Growth diagram of *S. aureus* Newman D2C (WT) in the presence or absence of CHA (256 μg/ml) and ΔSrtA.

Currently, research work in our group mainly focuses on finding new molecules from traditional Chinese medicine (TCM) against key virulence factors in bacteria. Inhibitors of the α-Hemolysin (Hla) of *S. aureus* and Listeriolysin O of *Listeria monocytogenes* have been reported recently (Qiu et al., 2012; Wang et al., 2014). By detecting the inhibition rate of enzyme activity, we have screened anti-Sa-SrtA molecules from TCM which have detoxification effects. Several compounds from medicinal herbs have been identified. We had a strong interest in chlorogenic acid (CHA) because of its relatively high inhibitory activities. CHA (**Figure 1A**) is a major component of *Flos Lonicerae*, which is one of the most common TCMs used for the treatment of various diseases including infections, fever, swelling, sores, and arthritis for 1000s of years (Wu, 2007). Pharmacological data obtained from *in vivo* and *in vitro* experiments show that CHA has a broad spectrum of biological activities, such as antibacterial, antioxidant, anti-inflammatory, antigenotoxic, anticancer and cytostatic activities and a relatively low toxicity, which indicates that CHA could be a potential leader drug (Niggeweg et al., 2004; dos Santos et al., 2006; Abraham et al., 2007; Chauhan et al., 2012; Weng and Yen, 2012). Although CHA has an obvious inhibitory effect on the growth of both Gram-negative and Gram-positive bacteria (Sung and Lee, 2010; Lou et al., 2011; Li et al., 2014), the minimum inhibitory concentrations (MICs) are relatively high. Therefore, the antibacterial activity of CHA would not be a reasonable explanation for its treatment of infectious diseases.

We systematically study herein the activity of CHA as a novel class of Sa-SrtA inhibitors *in vitro*. Moreover, we assessed the potential therapeutic effect of CHA on *S. aureus*- induced renal abscesses in a mouse model of infection. The mechanism of inhibition was further investigated using molecular dynamics simulations and conducting preliminary structure–activity relationship studies. To our knowledge, this is the first example of a SrtA inhibitor from a natural product to be evaluated for *in vitro* activity, adaptation symptoms and the molecular mechanism of interaction with the active site of the enzyme.

## Materials and Methods

### Bacterial Strains, Plasmids, and Growth Conditions

The bacterial strains and plasmids used in the present study are described in **Table 1**. The ΔSrtA strain was constructed from Newman D2C in the previous study by our team (Chen et al., 2013). *Escherichia coli* strain was grown in Luria-Bertani broth (LB) at 37°C while shaking and was supplemented, if required, with ampicillin (100 μg/mL). *S. aureus* strains were grown in brain-heart infusion (BHI) broth (Sigma) at 37°C under vigorous shaking and were supplemented, when required, with erythromycin (Erm, 2.5 μg/mL) and chloramphenicol (15 μg/mL). *S. aureus* recombinant SrtA was purified from *E. coli* strain BL21 (DE3) using GST affinity chromatography. Fluorescent peptide Dabcyl-QALPETGEE-Edans was synthesized by GL Biochem (Shanghai, China). The compound CHA was purchased from the National Institutes for Food and Drug Control of China.

**Table 1:** Strain and plasmid list.

Strain or plasmid	Relevant genotype	Reference
**Strains**
***Staphylococcus aureus***		
Newman D2C	wild-type SrtA positive; non-hemolytic; coagulase negative	ATCC25904
ΔSrtA	srtA::Erm; isogenic mutant of Newman D2C	Chen et al., 2013
***Escherichia coli***		
BL21	Expression strain, F^-^ ompT hsdS(rB^-^ mB^-^) gal dcm (DE3)	Invitrogen
**Plasmids**
pGEX-6P-1	Expression vector	Amersham
pGSrtAΔ*N_59_*	pGEX-6P-1 with *srtA* gene	This study
C184A	pGSrtAΔ*N59* derivative, for the substitution of Cys184 with alanine	This study
G192A	pGSrtAΔ*N59* derivative, for the substitution of Gly192 with alanine	This study

### Construction of SrtAΔ*N59* and Mutant Protein Expression Vectors

Primers designated as PsrtA59F and PsrtA59R were used to amplify a SrtAΔ*N59* sequence (which would express only residues 60–206) from genomic DNA from *S. aureus* NewmanD2C, and they were cloned into the pGEX-6P-1 vector to generate the pGSrtAΔ*N59* construct. Site- directed mutagenesis for C184A and G192A was performed as described in the stratagene protocol (TransGen Biotech) using pGSrtAΔ*N59* as the template. The complementary forward and reverse primer pairs employed to construct the SrtAΔ*N59* variants are listed in **Table 2**. All expression vectors were confirmed by DNA sequencing.

**Table 2:** Oligonucleotide primers used in this study.

Primer name	Oligonucleotide (5–3)^a^
PsrtA59F	GCGGGATCCCCGGAATTCCAAGCTAAACCTCAAATTCC
PsrtA59R	CCGCTCGAGTTATTTGACTTCTGTAGCTACAA
C184A–F	GCCTGTCTTTTCATTGTAATCATCAGCAGTAATTAATGTT
C184A–R	GCTGATGATTACAATGAAAAGACAGGCGTTTGGGAAAAAC
G192A–F	TATTTAATTGTTCAGCTGTTGCTGGT
G192A–R	GCTGAACAATTAAATAGAGGTGTAA

*^a^ Restriction endonuclease recognition sites or mutated codons are underlined*.

### Expression and Purification of SrtAΔ*N59* and Mutant SrtA

The pGSrtAΔ*N59* and mutant constructs were transformed into *E. coli* strain BL21 (Invitrogen, Carlsbad, CA, USA). The transformed cells were grown in LB broth with ampicillin at 37°C until the OD_600_ reached 0.6∼0.8. The culture was then induced with 1 mM isopropyl *â*-D-1-thiogalactopyranoside (IPTG, Invitrogen, Carlsbad, CA, USA) and grown overnight at 16°C. The cells were harvested and resuspended in the reaction buffer (Tris-HCl 50 mM, CaCl_2_ 5 mM, NaCl 150 mM, pH 7.5). After sonication, the lysate was centrifuged, and the supernatant was applied to a self-packaged GST-affinity column (2 mL glutathione Sepharose 4B; GE Amersham). After washing off the unbound contaminating proteins, the GST -tagged protein was digested with Precision Protease at 4°C overnight and then was eluted with reaction buffer. All samples were analyzed by SDS-PAGE for the presence of the recombinant protein, and its concentration was determined using the BCA protein assay kit (Pierce).

### Determination of SrtA Activity

The activity of CHA against SrtAΔ*N59* was determined using a fluorescence resonance energy transfer (FRET) assay. In this assay, the IC_50_ value was determined by monitoring the effect of CHA on the steady state cleavage of a model substrate peptide, Dabcyl-QALPETGEE-Edans. FRET assay protocols have been described previously (Ton-That et al., 1999; Mazmanian et al., 2002). Briefly, 300 μl of a mixture containing reaction buffer, recombinant SrtA 4 mM, and increasing concentrations of CHA was added to the 96-well plate and incubated for 30 min at 37°C. Then, the assays were started with the addition of the substrate modified peptide and run for 1 h at 37°C. The sample fluorescence was measured using emission and excitation wavelengths of 495 and 350 nm, respectively. Each experiment was repeated at least three times to ensure reproducibility.

### Determination of the MIC and Plotting of the Growth Curves

The MIC of CHA was determined by broth microdilution according to the NCCLS guideline M_31_-A_2_. CHA was dissolved in dimethyl sulfoxide (DMSO) and diluted with sterilized distilled water before use (final 0.5% DMSO, which was found to have no effect on enzyme activity). For Growth Curve Plotting, 1 mL of overnight bacterial cultures of *S. aureus* were added to 50 mL of fresh BHI broth and incubated at 37°C with or without CHA for different lengths of time. 0.5% DMSO was added to control cultures. The absorbency reading was taken at OD_600_.

### Fibrinogen-Binding Assay

The *S. aureus* wild-type strain was grown in BHI broth to the exponential phase using incubation in a shaking incubator at 37°C, diluted to an initial OD (600 nm) of 0.05, and with different concentrations of CHA added. The Newman ΔSrtA strain was grown under the same conditions as the positive control. All of the samples were cultivated for 2 h on a rotary shaker at 37°C. The cells were pelleted by centrifugation (5,000 × *g* for 5 min), washed twice and resuspended in PBS to an OD_600_ of 1.0.

Polystyrene Costar 96-well plates were coated overnight at 4°C with 100 μl of a 20 μg/ml bovine Fibrinogen (Fg). Plates were washed and blocked for 2 h at 37°C with BSA. After washing with PBS, 100 μl of cell suspension was added and incubated for 2 h at 37°C. The cell suspension was removed. The adherent bacterial cells were fixed with 25% (v/v) formaldehyde for 30 min after washing, and 100 μl crystal violet dye (12.5 g/l) was added and incubated for 10 min. The plate was washed again and dried, and then the absorbance of the plate was subsequently read at 570 nm with a Microplate reader. The results were reported as the percentage of the adherence rate compared to the wild type control. Each experiment was repeated at least three times to ensure reproducibility.

### Western Blot Analysis

Solubilized cell wall proteins and the cytoplasmic membrane were obtained as previously described (Hartford et al., 1997; Mazmanian et al., 2000). The protein extracts were separated on 12% SDS-PAGE gels and transferred to polyvinylidine difluoride membranes (Wako Pure Chemical Industries, Ltd., Osaka, Japan). The antibodies against *S. aureus* surface protein A was purchased from Abcam, and against *S. aureus* SrtA was prepared by our team members. HRP-labeled goat anti-chicken Igγ was purchased from Santa Cruz, and HRP-labeled goat anti-rabbit IgG was purchased from Proteintech.

### Animal Experiments

All experimental animals used in these studies were 6- to 8-weeks-old female BALB/c mice obtained from the Experimental Animal Center of Jilin University. The animal experiments were approved by and conducted in accordance with the guidelines of the Animal Care and Use Committee of Jilin University.

Overnight cultures of *S. aureus* were inoculated 1:100 into fresh BHI broth and grown for 3 h at 37°C. Staphylococci were washed with PBS twice and suspended in BHI broth. Bacteria (2 × 10^9^ CFU) were injected into the tail vein for survival studies. For the kidney infection model, BALB/c mice (*N* = 8 per group) were inoculated with 200 μl of staphylococcal suspension (2 × 10^8^ CFU) into the tail vein. On the 6th day after infection, the mice were euthanized and the kidneys were excised. The left kidneys were homogenized, diluted in normal saline and plated in duplicate for the determination of CFUs. The right kidneys from each group of mice were fixed in 10% formalin for 24 h at room temperature. The tissues were embedded in paraffin, thin-sectioned, stained with hematoxylin and eosin (H&E), and examined by microscopy. All animal studies were performed at least twice, and similar results were observed in all replicate experiments.

### Statistical Analysis

The statistical significance of the survival studies was assessed using Log-rank (Mantel-Cox); the significance of bacterial burden and percentage of Fg-binding were calculated using the unpaired two-tailed Student’s *t*-test. The differences were considered statistically significant when *P* < 0.05.

The other materials and methods used are described in the Supplementary materials section.

## Results

### CHA Inhibits the Activity of SrtA

The inhibitory activities from major components of detoxifying herbs on SrtA were determined using a FRET assay (Ton-That et al., 1999; Mazmanian et al., 2002; data not show). CHA, the main component of *Flos Lonicerae*, had a high activity relative to other tested compounds. Structurally, CHA is an ester formed between quinic acid and *trans*-cinnamic acid, which represents a new class of SrtA inhibitor (**Figure 1A**). The percentage of inhibitory activity of CHA (different concentrations) against SrtA is shown in **Figure 1B**. We determined that CHA exhibited potent sortase-inhibitory activity with IC_50_ 33.86 ± 5.55 μg/ml.

### CHA has No Influence on *S. aureus* Growth

The MIC values of CHA against tested *S. aureus* strains (*S. aureus* ATCC25904, *S. aureus* ATCC25923, and *S. aureus* ATCC29213) were all greater than 1024 μg/ml. We made a growth curve for *S. aureus* ATCC25904 to determine if the growth time changed when the CHA (256 μg/ml) was added to the BHI broth. We observed that the growth rate of *S. aureus* WT + CHA and ΔSrtA was similar to WT even the dosage is eight times its IC_50_ (**Figure 1C**). The results indicate that CHA could be a potential anti-virulence molecule which could effectively inhibit SrtA activity at a concentration far lower than the MIC.

### CHA Inhibits the Adhesion of *S. aureus* to Fibrinogen

**FIGURE 2:**
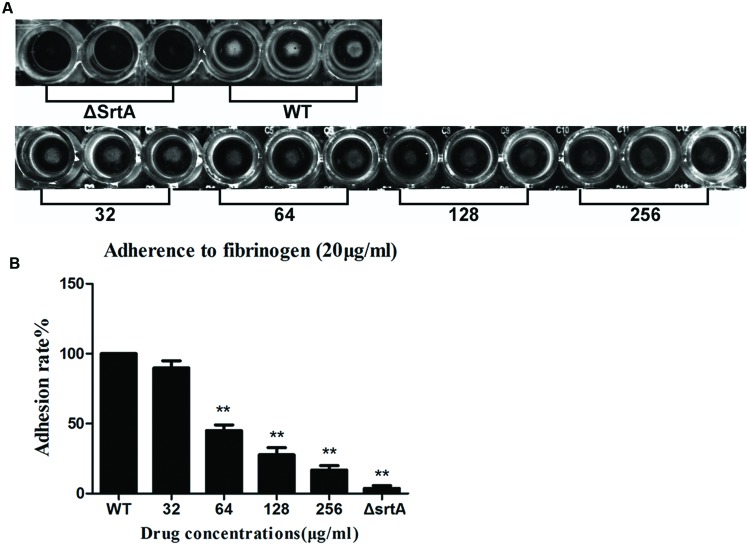
**Fibrinogen (Fg)-binding assays**. The value of the ΔSrtA as the ideal sortase inhibitor was used as a positive control. **(A)** The results could be inspected directly. **(B)** Adhesion rate of bacterial cells to Fg. CHA reduced the adhere of WT to Fg in a dose dependent manner. Each result was derived from three independent experiments and presented as the mean ± SEM. ^∗∗^*P* < 0.01 vs. the WT group.

According to the preceding conclusions, CHA has considerable inhibitory activity against SrtA. *S. aureus* can express up to 21 different surface proteins, such as clumping factors (ClfA and ClfB) and fibronectin-bining proteins A and B (FnbA and FnbB), many of which are covalently anchored to the cell wall by the catalysis of sortase transpeptidase (Roche et al., 2003; DeDent et al., 2008). Binding Fg and Fn are important for the pathogenesis of *S. aureus*. *S. aureus* mutants lacking *srtA* will fail to process and display surface proteins and are defective in the establishment of infections (Mazmanian et al., 2000). It can be inferred that CHA could interfere in Fg/Fn binding and attenuate the virulence of *S. aureus*. Therefore, we employed Fg-binding assays to test this hypothesis, in which cell adhesion to Fg-coated plates was quantified by measuring the absorbance following staining with crystal violet. First, the capacity of *S. aureus* strain Newman D2C (WT) and its isogenic knockout mutant ΔSrtA to adhere to Fg-coated surfaces was investigated. As shown in **Figures 2A,B**, the ΔSrtA showed a minimum binding rate to Fg, 3.7 ± 2.1%. The treatment of the WT strain with either 32, 64, 128, or 256 μg/ml of CHA was measured, and the adhesion rates were 89.8 ± 5.2, 45.0 ± 4.3, 27.7 ± 5.1, and 16.7 ± 3.2% to Fg, respectively (**Figure 2B**). We observed that the treatment of the WT strain with CHA reduced the capacity of the bacterium to adhere to Fg in a dose-dependent manner. When treating with 64, 128, 256 μg/ml of CHA, the binding was significantly reduced compared with that of the WT (**Figure 2B**).

### CHA Inhibits Transpeptidation Catalyzed by SrtA

**FIGURE 3:**
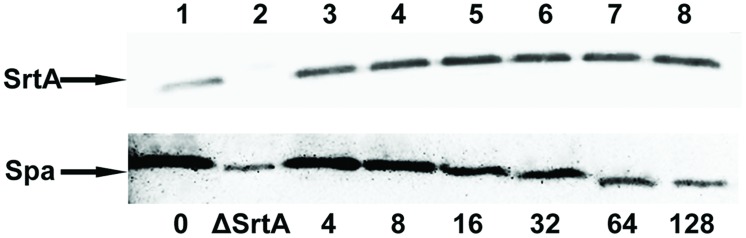
**Western blot analysis of sortase A (SrtA) protein and protein A (Spa)**. Proteins released from the cytoplasmic membrane (SrtA) or cell wall (Spa) of *S. aureus* Newman D2C (lanes 1, 3–8) and ΔSrtA (lane 2) grown in the absence or in the presence of CHA were fractionated by SDS-PAGE, transferred to polyvinylidene fluoride membranes. SrtA was detected with specific anti-SrtA rabbit antibodies and Spa was detected with specific anti-protein A chicken antibodies.

To determine whether CHA interferes with the function of surface proteins by reducing the amount of protein functionally displayed on the cell wall, we extracted cell wall-associated proteins. In this study, we demonstrated by Western blot analysis using anti-SrtA antibody that CHA does not influence the expression of SrtA. When performing Western blot analysis of protein A in cell wall-associated extracts using anti-protein A antibody, we found that protein A decreased remarkably after incubating *S. aureus* with CHA (**Figure 3**). These results demonstrated that CHA could be considered as an inhibitor to directly affect SrtA *in vivo*.

### Determination of the Binding Mechanism of SrtA with CHA

**FIGURE 4:**
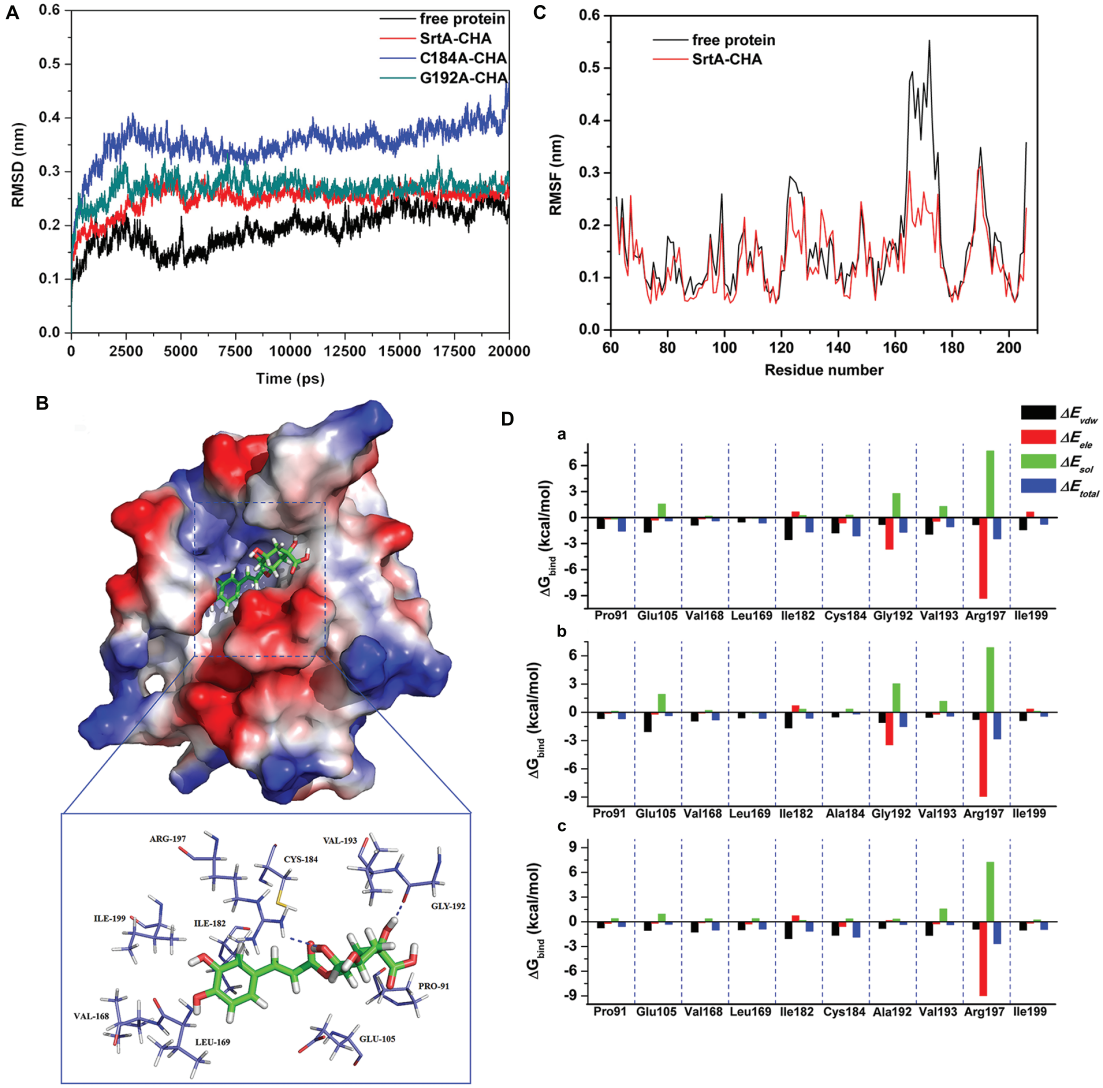
**(A)** The root-mean-square deviations (RMSDs) of all the atoms of SrtA-CHA complexes with respect to their initial structures as function of time. **(B)** The predicted binding mode of CHA in SrtA binding pocket obtained from MD simulation. **(C)** RMSF of residues of the whole protein in SrtA-CHA complex and free SrtA during the last 10-ns simulation time. **(D)** Decomposition of the binding energy on a per-residue basis in the SrtA-CHA complex (a), C184A-CHA complex (b), and G192A-CHA complex (c).

To explore the potential binding mechanism of CHA to SrtA, molecular docking and molecular dynamics simulations were performed using the AutoDock 4.0 and Gromacs 4.5.1 software package. The initial structure of SrtA was obtained from the X-ray structure (PDB code: 1T2P; Zong et al., 2004). The preferential binding mechanism of SrtA with CHA was determined by 20-ns molecular dynamics simulations based on the docking results. To explore the dynamic stability of the models and to ensure the rationality of the sampling strategy, the root-mean-square deviation (RMSD) values of the protein backbone based on the starting structure along the simulation time were calculated and plotted in **Figure 4A**. As shown in **Figure 4A**, the protein structures of all of the systems were stabilized during the simulations.

In the simulation, CHA represents a ligand that can bind to SrtA via hydrogen bonding and hydrophobic interactions. Over the time course of the simulation, CHA localized to the “activity” region, which is reported to participate in reactivity and is important for SrtA (Ton-That et al., 1999, 2000). The predicted binding mode of CHA with SrtA is illustrated in **Figure 4B**, and the electrostatic potentials of the residues around the binding site were mapped using APBS software (Baker et al., 2001). In detail, the binding model of CHA with the activity region of SrtA (**Figure 4B**) revealed that the hydroxyl group of the cyclohexane of CHA formed hydrogen bonds with both the side CHA in amino of Arg197 and the backbone of Gly192. Moreover, the side chains of Cys184 and Ile182 can form Van der Waals interactions with CHA, as shown in **Figure 4B**, which was confirmed by energy decomposition analysis.

The root mean square fluctuations (RMSF) of the residues of the whole protein in the SrtA-CHA complex and in free SrtA were calculated to reveal the flexibility of these residues. The RMSF of these residues are shown in **Figure 4C**, clearly depicting different flexibilities in the binding site of SrtA in the presence and absence of CHA. All of the residues in the SrtA binding site that bind with CHA show a small degree of flexibility with a RMSF of less than 3.00 Å when compared with free SrtA, indicating that these residues seem to be more rigid as a result of binding to CHA.

The above information indicated that the stabilization at the binding cavity of SrtA in this complex was mostly due to residues Ile182, Cys184, Gly192 and Arg197, as is shown in **Figure 4B**.

### Identification of the Binding Site in the SrtA-CHA Complex

To gain more information about the residues surrounding the binding site and their contribution to the whole system, the electrostatic, Van der Waals, solvation, and total contribution of the residues to the binding free energy were calculated with the Molecular MeCHAnics Generalized Born Surface Area (MM-GBSA) method (Punkvang et al., 2010; Schaffner-Barbero et al., 2010). The calculation was performed over the 100 MD snapshots taken from the last 10-ns simulation. The summations of the per residue interaction free energies were separated into Van der Waals (Δ*E_vdw_*), electrostatic (Δ*E_ele_*), solvation (Δ*E_sol_*), and total contribution (Δ*E_total_*). The energy contributions from the selected residues are summarized in **Figure 4D**-a. As shown, in the SrtA-CHA complex, Arg197 and Gly192 have an appreciable electrostatic (Δ*E_ele_*) contribution, with a Δ*E_ele_* of ≤-3.0 kcal/mol (**Figure 4D**-a). In fact, Arg197 and Gly192 are close to the cyclohexane group of CHA, and two electrostatic interactions exist, leading to two strong H-bonds between SrtA and CHA. In addition, residue Ile182 and Cys184, with a Δ*E_vdw_* of ≤-2.0 kcal/mol, have strong Van der Waals interactions with the ligand because of the close proximity between the residues and CHA. Except for residues Gly192 and Arg197, the majority of the decomposed energy interaction originated from Van der Waals interactions, apparently through hydrophobic interactions in the formation of the SrtA-CHA complex. In addition, the total binding free energy for the SrtA-CHA complex and its detailed energy contributions calculated according to the MM-GBSA approach are summarized in **Table 3**. With the summation of the solute entropy term (∼6.7 kcal/mol), an estimated Δ*G_bind_* of -15.2 kcal/mol was found for CHA, suggesting that CHA can strongly bind to and interact with the binding site of SrtA.

**Table 3:** The calculated energy components, binding free energy (kcal/mol), binding constants, and the number of binding sites using the fluorescence spectroscopy quenching method in the WT-CHA, C184A-CHA, and G192A-CHA systems.

	TΔ*S* (kcal/mol)	Δ*G_bind_* (kcal/mol)	Binding constants *K*_A_ (1 × 10^5^) L⋅mol^-1^	*n*
WT-CHA	6.7 ± 2.0	-15.2 ± 2.4	8.9 ± 1.3	0.9987
C184A-CHA	5.9 ± 1.4	-7.9 ± 1.6	4.7 ± 0.8	1.0025
G192A-CHA	6.3 ± 1.3	-8.4 ± 1.9	5.0 ± 0.9	0.9995

To examine the accuracy of the binding site in the SrtA-CHA complex, the mutant complexes of C184A-CHA and G192A-CHA were used as preliminary structures for MD simulations, and the MD trajectories were successively analyzed with the MM-GBSA method. The C184A and G192A mutants were expressed and purified, and the binding constants and number of binding sites between CHA and the two mutants were investigated by the fluorescence spectroscopy quenching method (Lakowicz and Weber, 1973).

As shown in **Figure 4D**-b,c, CHA binds to the two mutants and the WT-SrtA similarly, which was confirmed by the pair interaction decomposition of the free energy. The major contribution to the free energy was from Ile182, Cys184, Gly192, and Arg197. However, the MM-GBSA calculation predicted that C184A and G192A bound more weakly to CHA than did the WT-SrtA (-7.9 kcal/mol for C184A and -8.4 kcal/mol for G192A), as shown in **Table 3**. The calculations for C184A and G192A revealed that these mutants resulted in a decrease of ∼7 kcal/mol of binding energy compared to WT-SrtA. According to the experimental results, the binding constants, *K_A_*, of the interaction between CHA and SrtA decrease in the following order: WT > G192A > C184A, which means that WT-SrtA has the strongest ability to bind to CHA and C184A has the weakest ability, as shown in **Table 3**. The calculated binding free energies are in agreement with the experimental data. We believe that the MD simulations generated a reliable SrtA-CHA complex.

The residues of Ile182, Cys184, Gly192, and Arg197 have a key role in the binding process of SrtA with the substrate (Ton-That et al., 1999, 2000). Due to the binding of CHA with the activity region (residues of Ile182, Cys184, Gly192, and Arg197), the biological activity of SrtA was inhibited.

### The Effect of CHA on the Survival Rate of Mice Inoculated with *S. aureus*

**FIGURE 5:**
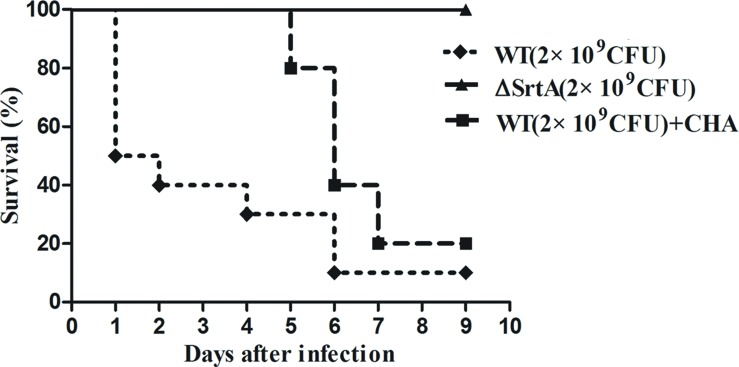
**Effects of CHA on survival rates after 9-days monitoring of BALB/c mice**. Survival percentage of BALB/c mice (*N* = 10) after challenged with intravenous injection of 2 × 10^9^ CFU of *S. aureus* (WT) and ΔSrtA. Treatment with CHA (50 mg/kg, three times a day) was initiated after inoculation. Survival statistics were calculated by Log-rank (Mantel–Cox) test. The statistical significance determined as follows: WT vs. ΔSrtA, *P* < 0.0001; WT vs. WT+CHA, *P* < 0.05.

Based on the *in vitro* findings above, we further investigated the protective effects of CHA *in vivo*. According to our previous study, 2 × 10^9^ CFU and 2 × 10^8^ CFU of *S. aureus* was injected during the survival and renal abscess experiments, respectively (Wang et al., 2015). Nine days after infection with 2 × 10^9^ CFU of *S. aureus*, the WT strain had produced significantly greater mortality, killing 90% of the mice within 7 days, compared with ΔSrtA strains, in which the mortality rate was 0% (**Figure 5**). WT-infected mice received a hypodermic injection of CHA at doses of 150 mg/kg/d. The survival analyses revealed that the WT+CHA group exhibited a significant survival advantage, particularly at early time points post-infection (**Figure 5**). The mice of the WT+CHA group started to die on day 5 post-injection (the day we stopped administrating the drugs). These results indicated that CHA can prolong survival and protect mice from death early in infection.

### CHA Protects Mice from *S. aureus*-Induced Renal Abscess

**FIGURE 6:**
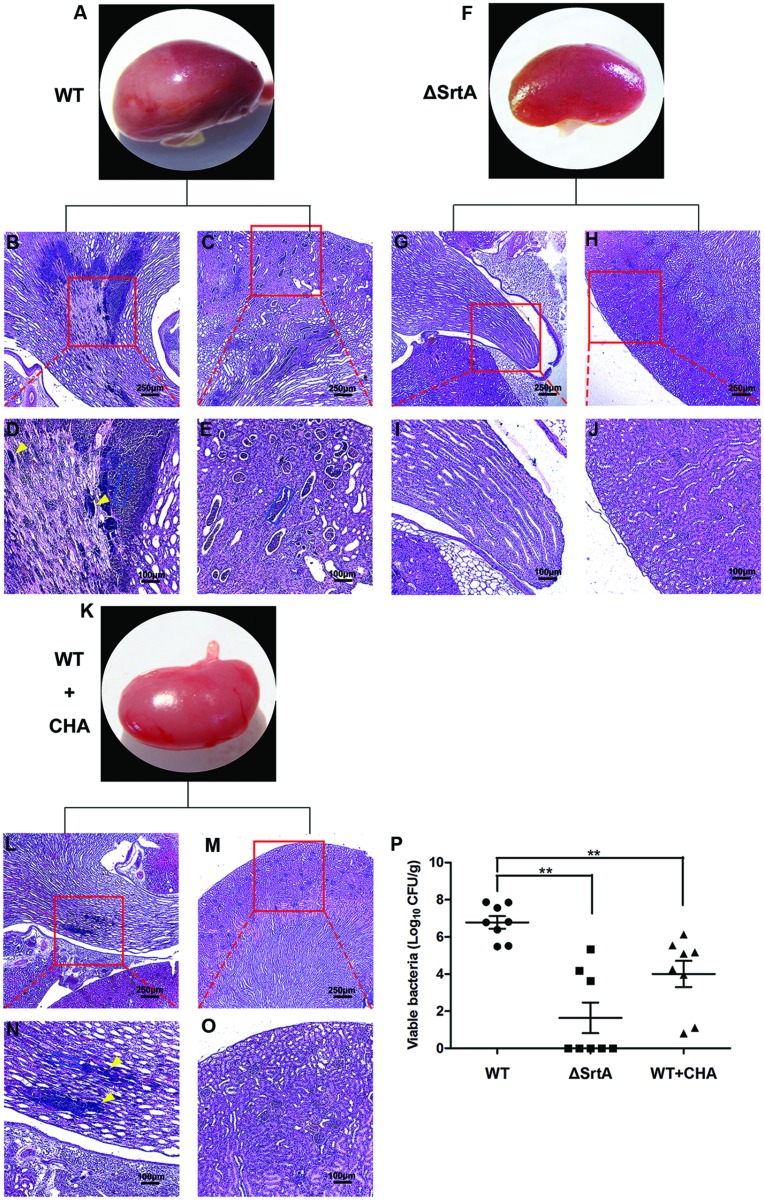
**Chlorogenic acid protects against *S. aureus*-induced abscess formation in kidney of BALB/c mice**. Kidneys of BALB/c mice (*N* = 8 per group) infected with 2 × 10^8^ CFU *S. aureus* Newman D2C (WT), ΔSrtA or WT after treatment with CHA (50 mg/kg, three times a day) were removed from mice on the 6 days after intravenous infection. **(A–P)** Kidneys were inspected for surface abscesses **(A,F,K)** or fixed in formalin, embedded, thin sectioned and stained with H&E. Histopathology images were acquired with light microscopy at ×100 **(B,C,G,H,L,M)** and ×400 **(D,E,I,J,N,O)**. Kidneys from mice challenged with WT had a big size of abscesses and enclosed a central population of staphylococci (**D**, yellow arrowheads), surrounded by a layer of eosinophilic, amorphous material and a large cuff of polymorphonuclear leukocytes (PMNs; **D,E**, blue rectangular box). There is no abscess founded in kidneys from mice infected with SrtA. Sizes of abscesses were significantly decreased after treated with CHA. Only some slight influx of PMNs (N, blue rectangular box) existed and harbored a few discernable organization of staphylococci (N, yellow arrowheads). **(P)** Bacterial burden in kidney tissue was measured on the 6 days after intravenous infection. Mean ± SEM of staphylococcal load were calculated as log_10_ CFU/g in homogenized renal tissues. Statistical significance was calculated with the Student’s *t*-test; ^∗∗^*P* < 0.01 vs. the WT group.

Following entry into the bloodstream, *S. aureus* escapes phagocytic killing by immune cells and, after binding to specific tissues, causes abscesses in organs (Lee et al., 1987). Surface proteins such as ClfA and FnbpA, which are displayed by SrtA, play an important role in this process (Cheng et al., 2009). To test the effect of CHA, a potential SrtA inhibitor, on the pathogenesis of staphylococcal diseases, we investigated the formation of renal abscesses in a mouse model of infection. In this model, 2 × 10^8^ CFU of *S. aureus* was used for infection (Wang et al., 2015). CHA (150 mg/kg/d) was hypodermically injected post-injection of *S. aureus*. The histopathology analysis of the kidneys from animals infected for 24 h revealed no appearance of abscesses on organ surfaces. The number of kidneys with surface abscesses increased to 25% by 72 h and to 70% on day 6. Six days after infection, the mice were euthanized and the kidneys were excised. The left kidneys were homogenized for determination of the staphylococcal load in renal tissue. We observed a mean of 6 × 10^6^ CFU/g of renal tissue for *S. aureus* Newman D2C, 40 CFU/g for *S. aureus* ΔSrtA (*P* < 0.01 vs. the WT group) and 10^4^ CFU/g for the CHA-treated group (*P* < 0.01 vs. the WT group; **Figure 6P**). After observing surface abscesses by microscopy at 10×, the right kidneys were fixed in 10% formalin. The tissues were embedded in paraffin, thin-sectioned, stained with hematoxylin and eosin (H&E), and examined by microscopy. As shown in **Figure 6**, the kidneys from mice challenged with WT had abscesses covering a large surface area (>50% of Surface; **Figure 6A**) and enclosed a central population of staphylococci, surrounded by a layer of eosinophilic, amorphous material and a large cuff of polymorphonuclear leukocytes (PMNs; **Figures 6B–E**; Cheng et al., 2009). ΔSrtA failed to form abscess lesions on either macroscopic or histopathological examination (**Figures 6F–J**). Sizes of the abscesses were significantly decreased after treatment with CHA, only a slight influx of PMNs was observed and there were a few discernible organizations of staphylococci (**Figures 6K–O**). These results indicate that SrtA is an important factor in the *S. aureus*-mediated formation of renal abscesses. Furthermore, CHA prevents mice from forming *S. aureus*-induced renal abscess and relieves the infection.

## Discussion

The widespread occurrence of methicillin-resistant *S. aureus* (MRSA; Otto, 2012) and vancomycin-resistant strains (VRSA; Welsh et al., 2010) creates an urgent need for new therapeutic agents to treat *S. aureus* infections. Traditional antibiotics act by preventing the synthesis and assembly of key components of bacterial processes that are essential for growth, which results in substantial stress on the target bacterium and rapidly selects for resistant subpopulations. Searching for a virulence inhibitor is one of the alternative approaches to find molecules with radically new mechanisms of action to treat infections caused by multidrug resistant bacteria (Rasko and Sperandio, 2010). Because most virulence traits are not essential for bacterial growth, this strategy might decrease the development of resistance by not inducing selective pressures (Rasko and Sperandio, 2010).

The key virulence factors produced by *S. aureus* include surface adhesion proteins and extracellular toxins responsible for the colonization of and damage to mammalian hosts (Schlievert et al., 2000). Among ∼30 exoproteins that *S. aureus* produces, α-Hemolysin (Hla) is the only one that is necessary for the pathogenicity of this bacterium (Wardenburg et al., 2007). Therefore, Hla could be a potential anti-virulence target to treat *S. aureus* infections. However, this type of pore-forming cytotoxin is quite different in its structure and function among bacterial species (Gonzalez et al., 2008). SrtA mediates the covalent attachment of up to 19 surface proteins to the cell wall, and it is very important for the tissue colonization and infection of *S. aureus*. SrtA represents another promising anti-virulence target (Maresso and Schneewind, 2008). Unlike the pore-forming cytotoxin, SrtA is a “housekeeping” sorting enzyme, and its homologs are conserved and can be found in almost all Gram-positive bacteria (Papadopoulos and Agarwala, 2007). As a target candidate for the treatment of Gram-positive bacterial infections, SrtA has drawn much attention since its identification ∼15 years ago (Mazmanian et al., 1999).

The search for inhibitors of SrtA have involved natural, synthetic, and high-throughput screening methodologies, and several distinct inhibitor classes have been identified (Maresso and Schneewind, 2008; Clancy et al., 2010). Among the inhibitors, only two chemically synthesized small molecules had a protective effect against *S. aureus* infections *in vivo* (Zhang et al., 2014). Nevertheless, the molecular mechanism of the interaction between the small molecules and enzyme remain unclear.

We have tried to find potent inhibitors from a number of natural small molecule compounds. CHA, an ester of caffeic and quinic, belongs to the phenylpropanoids and is quite structurally different from the known SrtA inhibitors (**Figure 1A**). At a concentration largely below the MIC, CHA could significantly inhibit the catalytic activity of SrtA *in vitro*, which means that the compound would be able to reduce the virulence of the pathogenic bacteria without exerting a noticeable selective pressure. We also evaluated the *in vivo* effects of CHA in a BALB/c mouse model. The results indicated that CHA could inhibit SrtA activity in mice, thereby causing a reduction in both the mortality rate and the formation of renal abscesses.

The wild type *S. aureus* that we used in this study was Newman D2C, which has been reported as a new reference strain for SrtA studies (Miller et al., 1977) due to its being coagulase-negative (Yonemasu et al., 1987, 1991) and non-hemolytic (Chen et al., 2013). To our current knowledge, SrtA is the most important virulence factor in this strain, and therefore this strain has an advantage for studying the function of SrtA and the activity of SrtA inhibitors. Consequently, a *srtA* mutant strain, ΔSrtA, was constructed based on the wild-type Newman D2C in our previous research (Chen et al., 2013).

Chlorogenic acid is a major component of *Flos Lonicerae*. The ancient herb has been used in the treatment of infectious diseases for 1000s of years (Wu, 2007). The oral and injectable preparations are also widely used in China currently (Clifford, 2000; Lou et al., 2011). The bioavailability and safety of CHA would be a prominent advantage over the synthesis inhibitors of SrtA that have been previously reported, although the IC_50_ of CHA is relatively high compared with those compounds.

Resent research demonstrated that CHA can also significantly down-regulate the expression level of enterotoxins (SEs) and *α-toxin*, which are important in the pathogenesis of *S. aureus* infections (Li et al., 2014). Taken together, through interference with both surface adhesins and exotoxins, CHA could have multi-faceted anti-virulence activity. It also could be more difficult for bacteria to develop resistance to these actions of CHA.

In this study, we also revealed the binding mechanism of CHA with SrtA by the molecular simulation method. The results indicated that CHA tightly occupied the active site of SrtA. According to the previous study, the β6/β7 loop, β7/β8 loop and the active residues Arg197, H120, and C184 are the key structural elements involved in the catalysis reaction (Suree et al., 2009). The mobile β6/β7 loop plays an important role in the substrate-specific recognition and catalysis of SrtA (Bentley et al., 2007, 2008). The aromatic ring of CHA is placed in the sorting signal recognition region of SrtA through hydrophobic interactions with the side chains of residues from the β6/β7 loop (Val169, Leu168). The RMSF comparison of the residues of the apo-SrtA and CHA-SrtA complexes revealed that CHA significantly immobilizes the flexible β6/β7 loop, similar to the conformational changes induce by the binding of natural substrates. Thus, the binding of CHA would substantially interfere with substrate recognition, the initial step of the transpeptidation reaction of SrtA. Arg197 is essential in the catalysis of SrtA by stabilizing the positioning of the substrate peptide and stabilizing the tetrahedral intermediates of catalysis through interaction between its side chain and the oxyanion. In the docking model of CHA-SrtA, the guanidine group of Arg197 formed H-bonds with the –OH of the cyclohexane moiety of CHA. Through the hydrophobic interactions between the side chains of Cys184 and Ile 182 and the central region of CHA, the complex was further stabilized in the active site. Upon binding of the natural substrate peptide, the β7/β8 of SrtA undergoes a dramatic conformation change and creates a new groove, which serves as a binding site for Lipid II. The hydroxyl group of the cyclohexane, which occupies the Lipid II site of SrtA, forms another hydrogen bond with the backbone carbonyl of Gly192 and keeps the β7/β8 loop in a closed state in the CHA-SrtA complex.

This evidence suggests that, through preventing the access and binding of the SS (sorting signal) of the surface protein to the active site, CHA effectively inhibits the transpeptidation of SrtA. This would be critical information for further reasonable design of more powerful inhibitors. We are currently conducting studies regarding structural modifications of CHA and the evaluation of its inhibition against SrtA from *S. aureus* and other Gram-positive bacteria.

## Conclusion

We have determined that CHA is a novel SrtA inhibitor which is quite different structurally from the chemical moieties reported previously. *In vivo*, CHA significantly interferes in the pathogenesis of *S. aureus* and provides protection against renal abscess formation in murine models. For the first time, the detailed interactions between SrtA and the inhibitor were analyzed. This should pave the way for the generation of CHA derivatives with higher potency against SrtA and lead to the development of effective and clinically useful anti-virulence agents.

## Author Contributions

XD and DW designed the study. LW, CB and HC performed the *in vivo* experiments. BL and XZ performed the *in vitro* experiments. XN performed the Molecular docking and dynamic simulation experiments. LW and CB wrote the manuscript. TW and HX edited and modified the manuscript.

## Conflict of Interest Statement

The authors declare that the research was conducted in the absence of any commercial or financial relationships that could be construed as a potential conflict of interest.
