# Supplementary material for: The therapeutic effect of chlorogenic acid against Staphylococcus aureus infection through sortase A inhibition
Source: Front Microbiol. 2015 Oct 16;6:1031. doi: 10.3389/fmicb.2015.01031 (PMC4608362; doi:10.3389/fmicb.2015.01031)
Supplement: Supplementary file 1 [file Presentation_1.PDF]

Supplementary Material

Materials and Methods

Molecular docking calculation

The initial structure of SrtA was obtained from the 3D structure of X-ray. To obtain the starting structure of the ligand/SrtA complex for molecular dynamics (MD) simulation, a standard docking procedure for a rigid protein and a flexible ligand was performed with AutoDock 4 (Hu et al., 2010; Morris et al., 2013). The Lamarckian genetic algorithm (LGA) was applied in the docking calculations. All of the torsional bonds of the ligand were free to rotate while SrtA was held rigid. Then, the polar hydrogen atoms were added for SrtA using the AutoDock tools, and Kollman united atom partial CHArges (Morris et al., 1996) were assigned. A total of 150 independent runs were carried out with a maximum of energy evaluations to 25,000,000 and a population size to 300. A grid box (70×70×70) with spacing of 0.375 Å was created and centered on the mass center of the ligand. Energy grid maps for all possible ligand atom types were generated using Autogrid 4 before performing the docking.

The clusters were ranked according to the lowest energy representative in each cluster. The lowest energy conformation in the most populated cluster was chosen for further study (Hu et al., 2009).

Molecular Dynamics (MD) Simulations

All simulations and the analysis of the trajectories were performed using the Gromacs 4.5.1 software package (Hess et al., 2008) using the CHArmm27 force field and the TIP3P water model (Jorgensen et al., 1983). The SrtA-CHA system was first energy relaxed with 2000 steps of steepest-descent energy minimization followed by another 2000 steps of conjugate gradient energy minimization. The system was then equilibrated by a 500 ps of MD run with position restraints on the protein and ligand to allow relaxation of the solvent molecules. The first equilibration run was followed by a 20 ns MD run without position restraints on the solute. The first 10 ns of the trajectory were not used in the subsequent analysis in order to minimize convergence artifacts. Equilibration of the trajectory was checked by monitoring the equilibration of quantities, such as the root-mean-square deviation (RMSD) with respect to the initial structure, the internal protein energy, and fluctuations were calculated on different time intervals. The electrostatic term was described by using the particle mesh Ewald algorithm. The LINCS (Ryckaert et al., 1997) algorithm was used to constrain all bond lengths. For the water molecules, the SETTLE algorithm (Berendsen et al., 1984) was used. A dielectric permittivity, $\varepsilon = 1$, and a time step of 2 fs were used. All atoms were given an initial velocity obtained from a Maxwellian distribution at the desired initial temperature of 300 K. The density of the system was adjusted performing the first equilibration runs at *NPT* condition by weak coupling to a bath of constant pressure ($P_0 = 1$ bar, coupling time $\tau_P = 0.5$ ps) (Wang et al., 2006). In all simulations, the temperature was maintained close to the intended values by

weak coupling to an external temperature bath with a coupling constant of 0.1 ps. The structural cluster analysis was carried out using the method described by Daura and co-workers with a cut off of 0.25 nm (Ryckaert et al., 1997).

The CHA parameters were estimated with the anteCHAmber programs (Wang et al., 2006) and AM1-BCC partial atomic CHArges from the Amber suite of programs (Jakalian et al., 2002).

Calculation of Binding Free Energy

In this work, the binding free energies are calculated using Molecular MeCHAnics Generalized Born Surface Area (MM-GBSA) approach supplied with Amber 10 package. We choose a total number of 100 snapshots evenly from the last 10 ns on the MD trajectory. The MM-PBSA method can be conceptually summarized as:

$$\Delta G_{bind} = \Delta G_{complex} - [\Delta G_{protein} + \Delta G_{lig}] \quad (1)$$

$$\Delta G_{bind} = \Delta H - T\Delta S \quad (2)$$

where ΔH of the system is composed of the enthalpy CHAnges in the gas phase upon complex formation (ΔE_{MM}) and the solvated free energy contribution (ΔG_{sol}), while $-T\Delta S$ refers to the entropy contribution to the binding. Eq. (2) can be then approximated as shown in Eq. (3):

$$\Delta G_{bind} = \Delta E_{MM} + \Delta G_{sol} - T\Delta S \quad (3)$$

where ΔE_{MM} is the summation of the van der Waals (ΔE_{vdw}) and the electrostatic (ΔE_{ele}) interaction energies.

$$\Delta E_{MM} = \Delta E_{vdw} + \Delta E_{ele} \quad (4)$$

In addition, ΔG_{sol}, which denotes the solvation free energy, can be computed as the summation of an electrostatic component ($\Delta G_{ele,sol}$) and a nonpolar component ($\Delta G_{nonpolar,sol}$), as shown in Eq. (5):

$$\Delta G_{sol} = \Delta G_{ele,sol} + \Delta G_{nonpolar,sol} \quad (5)$$

Ligand-residue Interaction Decomposition

The interactions between adenosine and each residue in the binding site of SrtA are analyzed using the MM-GBSA decomposition process applied in the MM-GBSA module in Amber 10. The binding interaction of each ligand-residue pair includes three terms: van der Waals contribution (ΔE_{vdw}), electrostatic contribution (ΔE_{ele}), and solvation contribution (ΔE_{sol}). All energy components are calculated using the same snapshots as the free energy calculation.

Binding affinity determination of CHA with WT-SrtA, C123A and G131A

The binding constants (K_A) of CHA to the binding site on WT-SrtA, C123A and G131A were measured using the fluorescence-quenching method. Fluorescence spectrofluorimetry measurements were carried out using a Horiba Jobin-Yvon Fluorolog 3-221 spectrofluorometer (Horiba Jobin-Yvon, Edison, NJ). The measurements were acquired using a 280-nm excitation wavelength with a 5-nm band-pass and a 345-nm emission wavelength with a 10-nm band-pass. Details of the measurements were described previously (Bandyopadhyay et al., 2002; Jurasekova et al., 2009).

References

Bandyopadhyay S., Valder C. R., Huynh H. G., Ren H. and Allison W. S. (2002). The beta G156C substitution in the F1-ATPase from the thermophilic Bacillus PS3 affects catalytic site cooperativity by destabilizing the closed conformation of the catalytic site. Biochemistry. 41, 14421-14429.

Berendsen H. J. C, Postma J. P. M., van Gunsteren W. F., DiNola A. and Haak J. R. (1984). Molecular dynamics with coupling to an external bath. J. Chem. Phys. 81, 3684–3690.

Hess B., Kutzner C., Van der Spoel D. and Lindahl E. (2008). GROMACS 4: algorithms for highly efficient, load-balanced, and scalable molecular simulation. J. Chem. Theory. Comput. 4, 435–447.

Hu R., Barbault F., Delamar M. and Zhang R. (2009). Receptor- and ligand-based 3D-QSAR study for a series of non-nucleoside HIV-1 reverse transcriptase inhibitors. Bioorg. Med. Chem. 17, 2400-2409.

Hu R., Barbault F., Maurel F., Delamar M. and Zhang R. (2010). Molecular dynamics simulations of 2-amino-6-arylsulphonylbenzonitriles analogues as HIV inhibitors: interaction modes and binding free energies. Chem. Biol. Drug. Des. 76, 518-526.

Jakalian A., Jack D. B. and Bayly C. I. (2002). Fast, efficient generation of high-quality atomic charges. AM1-BCC model: II. Parameterization and validation. J. Comput. Chem. 23, 1623–1641.

Jorgensen W. L., Chandrasekhar J., Madura J. D., Impey R. W. and Klein M. L. (1983). Comparison of simple potential functions for simulating liquid water. J. Chem. Phys. 79, 926–935.

Jurasekova Z., Marconi G., Sanchez-Cortes S. and Torreggiani A. (2009). Spectroscopic and molecular modeling studies on the binding of the flavonoid luteolin and human serum albumin. Biopolymers. 91, 917-927.

Morris G. M., Goodsell D. S., Huey R. and Olson A. J. (1996). Distributed automated docking of flexible ligands to proteins: parallel applications of AutoDock 2.4. J. Comput. Aided. Mol. Des. 10, 293-304.

Morris G. M., Huey R., Lindstrom W., Sanner M. F., Belew R. K., Goodsell D. S. et al. (2009). AutoDock4 and AutoDockTools4: Automated docking with selective receptor flexibility. J. Comput. Chem. 30, 2785-2791.

Ryckaert J. P., Ciccotti G. and Berendsen H. J. C. (1997). Numerical integration of cartesian equations of motion of a system with constrained molecular dynamics of N-alkanes. J. Comput. Phys. 23, 327–341.

Wang J., Wang W., Kollman P. A. and Case D. A. (2006). Automatic atom type and bond type perception in molecular mechanical calculations. J. Mol. Graph. Model. 25, 247–260.
